# Neural Correlates of Sex/Gender Differences in Humor Processing for Different Joke Types

**DOI:** 10.3389/fpsyg.2016.00536

**Published:** 2016-04-26

**Authors:** Yu-Chen Chan

**Affiliations:** Institute of Learning Sciences, National Tsing Hua UniversityHsinchu, Taiwan

**Keywords:** fMRI, sex/gender, verbal jokes, humor techniques, logical mechanisms, tri-component theory of humor

## Abstract

Humor operates through a variety of techniques, which first generate surprise and then amusement and laughter once the unexpected incongruity is resolved. As different types of jokes use different techniques, the corresponding humor processes also differ. The present study builds on the framework of the ‘tri-component theory of humor,’ which details the mechanisms involved in cognition (comprehension), affect (appreciation), and laughter (expression). This study seeks to identify differences among joke types and between sexes/genders in the neural mechanisms underlying humor processing. Three types of verbal jokes, bridging-inference jokes (BJs), exaggeration jokes (EJs), and ambiguity jokes (AJs), were used as stimuli. The findings revealed differences in brain activity for an interaction between sex/gender and joke type. For BJs, women displayed greater activation in the *temporoparietal–mesocortical-motor network* than men, demonstrating the importance of the temporoparietal junction (TPJ) presumably for ‘theory of mind’ processing, the orbitofrontal cortex for motivational functions and reward coding, and the supplementary motor area for laughter. Women also showed greater activation than men in the *frontal-mesolimbic network* associated with EJs, including the anterior (frontopolar) prefrontal cortex (aPFC, BA 10) for executive control processes, and the amygdala and midbrain for reward anticipation and salience processes. Conversely, AJs elicited greater activation in men than women in the *frontal-paralimbic network*, including the dorsal prefrontal cortex (dPFC) and parahippocampal gyrus. All joke types elicited greater activation in the aPFC of women than of men, whereas men showed greater activation than women in the dPFC. To confirm the findings related to sex/gender differences, random group analysis and within group variance analysis were also performed. These findings help further establish the mechanisms underlying the processing of different joke types for the sexes/genders and provide a neural foundation for a theory of sex/gender differences in humor.

## Introduction

Previous fMRI studies of humor have focused on segregating cognitive and affective processing (Goel and Dolan, 2001; Moran et al., 2004; Bartolo et al., 2006; Chan et al., 2012, 2013). A broad understanding of humor processing supporting different types of humor along with their neural correlates has emerged in recent years (Goel and Dolan, 2001; Watson et al., 2007; Samson et al., 2008, 2009; Bekinschtein et al., 2011; Chan and Lavallee, 2015). In our recent research, we identified the distinct neural correlates of the cognitive operations required to comprehend the logical mechanisms of three joke types: bridging-inference jokes (BJs), exaggeration jokes (EJs), and ambiguity jokes (AJs) (Chan and Lavallee, 2015). In a separate line of research, the neural correlates of humor processing between the sexes/genders have been a topic of interest for years (Azim et al., 2005; Kohn et al., 2011; Vrticka et al., 2013). To date, there has been no research, however, on the neural correlates of sex/gender differences in humor processing supporting particular types of jokes. Based on our earlier findings related to joke types (Chan and Lavallee, 2015), the present study is an attempt to further investigate the neural correlates of sex/gender differences underlying the humor processing of three types of jokes.

### Sex/Gender Differences and Theories of Humor

A number of sex/gender-based behavioral differences in humor production and appreciation have been observed. Most generally, men have been found to be more likely to produce humor, whereas women are more likely to act as an appreciative audience than to produce humor on their own (see Martin, 2007, p. 187, for a review and see Li et al., 2009; Vrticka et al., 2013). Women are more likely than men to be sexually attracted to a person who produces humor (Bressler and Balshine, 2006; Cooper et al., 2007), and men appear to put more effort into generating humor, particularly in mixed-sex contexts (Crawford and Gressley, 1991). When presented with descriptions of two individuals of the opposite sex and asked to choose which one was more attractive as a potential romantic partner, women tended to choose the one described as producing humor and making them laugh over the one who appreciated their humor, whereas men tended to favor the humor appreciator over the humor producer (Bressler et al., 2006).

These findings are broadly consistent with an evolutionary theory of sex/gender differences in humor, and indeed such theories have been proposed. The idea that humor may be attractive as a signal of genetic quality is rooted in Darwin’s (1871) sexual selection theory. From an evolutionary perspective, humor and laughter may have played key roles. People with a sense of humor were likely to have been popular because it was a signal for good genes, in that generating humor involves superior cognitive skills which would also generate advantages related to survival and reproduction (Martin, 2007, 2014). Preferred selection criteria for mates are thought to include markers of good genes and these markers may include displays of both humor creation and appreciation. Miller’s (1998, 2000) evolutionary theory of humor argued that sexual selection played a vital role in the evolution of humor in humans. Similarly, evolutionary neuroandrogenic theory (ENA theory) asserts that numerous gender differences in cognition and behavior, including humor, are best explained by evolutionarily-shaped genetic factors (Ellis, 2011).

However, the evidence in favor of such theories is far from conclusive. Earlier research based on the classical sexual selection hypothesis (e.g., Bateman, 1948) has been called into question (e.g., Okuda, 1999; Snyder and Gowaty, 2007; Gowaty et al., 2012, on Bateman’s measurement of fitness variance), and an alternative to the classical hypothesis, involving ‘sex-role reversal’ with female–female competition and choice by males has been proposed (e.g., Vincent et al., 1992; Eens and Pinxten, 2000; Zuk, 2002).

Sex/gender differences in humor appreciation have also been explained in terms of sociocultural factors (Zippin, 1966; Brodzinsky and Rubien, 1976; Weisfeld, 1993; Polimeni and Reiss, 2006; Vivona, 2014), situational context (Kotthoff, 2006) and personality-related differences (Zillmann and Cantor, 1976). Thus, throughout, we have used the term “sex/gender,” which emphasizes an intertwinement of biologically determined and socially acquired differences (Kaiser et al., 2009; Kaiser, 2012). In short, while the existence of sex/gender-based behavioral differences related to humor appears well established, the precise nature and causation of these differences is likely to remain an active area of research.

### Tri-component Theory of Humor

**FIGURE 1:**
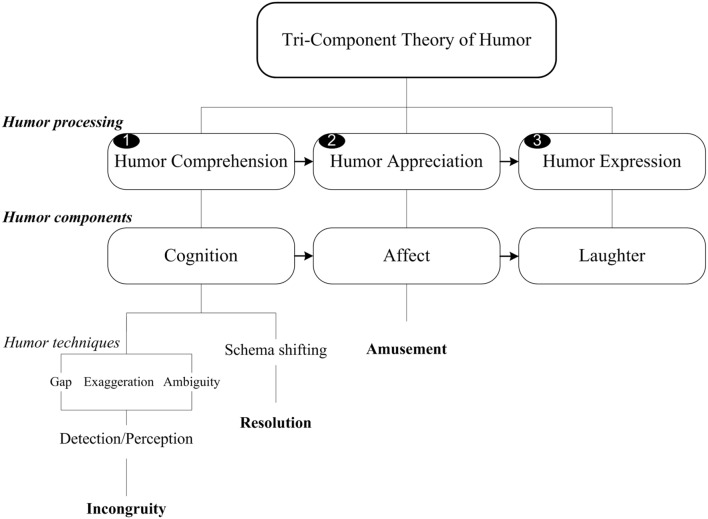
**Tri-Component Theory of Humor.** Humor processing is composed of humor comprehension (incongruity and resolution), humor appreciation (amusement), and humor expression (laughter). Humor components include cognition, affect, and laughter.

The present study builds on the framework of the ‘Tri-Component Theory of Humor,’ which details the mechanisms involved in humor comprehension (cognition), humor appreciation (affect), and humor expression (laughter) (**Figure 1**). This three-stage humor processing theory involves (1) the resolution of the central incongruity through schema shifting during the cognitive stage, (2) a positive feeling state related to amusement, mirth, or reward during the affective stage, and (3) the physical behavioral response to the positive emotion of amusement during the expressive stage. Note that within the model, the response to humor is divided into two distinct stages or components: amusement (implicit representation) and laughter (explicit behavior).

### Humor Techniques: Types of Jokes

Our analysis of different joke types is based on the General Theory of Verbal Humor (GTVH), which categorizes different types of verbal humor according to the ‘*logical mechanisms*’ that are required to resolve their central incongruity (Attardo and Raskin, 1991). Incongruity, a conflict between what is expected and what actually occurs, has long been held to be an essential component of humor (Shultz, 1976). The GTVH adds the further claim that verbal humor operates through a set of distinct techniques or ‘logical mechanisms,’ which are used to artfully construct incongruities that can be resolved in an amusing way. The use of different techniques calls upon correspondingly different cognitive processes for their comprehension. The ‘joke’ is commonly considered the fundamental form of verbal humor (Dynel, 2009). Different joke types employ different techniques (i.e., logical mechanisms), and the cognitive, affective, and expressive processes occurring in the listener differ correspondingly. Attardo et al. (2002) further classified the different types of logical mechanisms, and the present study examines three of the logical mechanisms identified in that study: inferring consequences, exaggeration, and juxtaposition.

#### Bridging-Inference Jokes (BJs)

Bridging-inference jokes, which are constructed using the ‘inferring consequences’ logical mechanism to construct semantic gaps, were defined as jokes in which the key point of the joke is not explicitly stated, and readers must use backward inferencing to understand the meaning and resolve the incongruity in an amusing way. As this typically requires inferences about the beliefs or attributing intentions of others, theory of mind (ToM) processing is likely involved in the resolution of such jokes.

#### Exaggeration Jokes (EJs)

Exaggeration jokes, which are constructed using the ‘exaggeration’ logical mechanism to generate semantic distortion, were defined as jokes in which the features or characteristics of some element of a situation are considerably bigger or smaller than expected, creating an incongruity.

#### Ambiguity Jokes (AJs)

Ambiguity jokes, which are constructed using the ‘juxtaposition’ logical mechanism to generate linguistic ambiguity, involve double meanings that require disambiguation for comprehension to occur. Verbal jokes often use linguistic ambiguity to create incongruity, and AJs are commonly used in verbally expressed humor that requires disambiguation (Attardo, 2001; Bekinschtein et al., 2011; Chan and Lavallee, 2015).

Contrasting the joke types, we can see that EJs involve semantic distortion whereas AJs involve linguistic ambiguity. BJs require the reader (or listener) to use inferences to fill a semantic gap and comprehend the joke. EJs and AJs, on the other hand, do not require inferencing to *comprehend* the joke but rather require the reader to make disparaging inferences about the target of the joke to *appreciate* the humor.

### Sex/Gender and Types of Jokes

Women and men display different patterns of humor preference in response to different types of humor (O’Connell, 1960; Wilson, 1975; Mundorf et al., 1988; Lampert and Ervin-Tripp, 1998; Martin, 2007, p. 143; Martin, 2014) and even different types of jokes (e.g., Groch, 1974). Interestingly, women and men appear to differ in humor appreciation not only in terms of the content of humor but also in terms of the preferred *structure* of humor (Derks and Arora, 1993). Behavioral studies of humor appreciation have generally indicated that men are more likely than women to enjoy humor containing aggressive or sexual *content*, whereas women are more likely to enjoy nonsensical or absurd humor *structures* (e.g., Groch, 1974; Terry and Ertel, 1974).

Specific differences related to the humor structure have been reported. Landis and Ross (1933) found that extroverted women tended to use exaggeration humor, whereas men of superior intelligence tended to use humor that involved projecting oneself into a situation. Khoury (1977) found that women were more likely than men to enjoy jokes based on the semantic technique of allusion (similar to the BJs discussed above). The study also found that jokes with double meanings (i.e., linguistic AJs) were funnier to men than women, but the difference was not statistically significant.

Additionally, women have been found to exhibit more smiles than men (Provine, 2001; LaFrance et al., 2003). Furthermore, the expressive behaviors of laughing and smiling have been found to directly influences the funniness ratings given by women but not by men (Cupchik and Leventhal, 1974).

### The Neural Correlates of Three Types of Jokes in Humor Processing

The neural correlates of the interaction between joke type and funniness have become a topic of major interest (Watson et al., 2007; Bekinschtein et al., 2011; Chan and Lavallee, 2015). In recent research, we employed event-related fMRI to examine the neural correlates of humor processing for three distinct types of verbal jokes: BJs, EJs, and AJs (Chan and Lavallee, 2015). The findings revealed differences in brain activity for an interaction between joke type and funniness.

The temporo-parietal lobe (TPJ and MTG) was specifically involved in processing BJs, with TPJ involvement likely reflecting involvement of these regions in ‘ToM’ processing for this type of joke. Social-affective appreciation of BJs was associated with activation in the orbitofrontal cortex (OFC). Additionally, the fronto-parietal lobe (IPL and IFG) was activated for both EJs and AJs, suggesting that it supports executive control processes, such as retrieval from episodic memory, self-awareness, and language-based decoding. The social affective appreciation of verbal jokes was associated with activation in the amygdala for EJs and parahippocampal gyrus (PHG) for AJs.

### The Neural Correlates of Sex/Gender Differences in Humor Appreciation

Previous studies have shown that the neural correlates of humor appreciation are different for women and men. Azim et al. (2005) found that women showed greater activation of the left prefrontal cortex and mesolimbic regions than men, suggesting a greater degree of executive processing and reward network response in women. (This study did not, however, find any region for which men showed significantly more activation than women.) Kohn et al. (2011) conducted an fMRI experiment using online subjective funniness ratings for parametric modulation. They found that the limbic system (amygdala, insula, and anterior cingulate cortex), was more active in women than in men. In contrast, men showed greater activation in the thalamus and in the dorsal processing system, including the dorsolateral prefrontal cortex (dlPFC) than women. The study further demonstrated that women process humor though limbic reactivation, involving an appraisal of its emotional features, whereas men apply more evaluative and executive resources to humor processing. Vrticka et al. (2013) used funny versus positive and neutral video clips with children. Girls exhibited significantly greater activation in the midbrain, amygdala, and temporo-occipital cortex in response to the funny clips, whereas boys showed significantly greater activation in the bilateral inferior parietal lobule (IPL), fusiform gyrus, inferior frontal gyrus (IFG), amygdala, and ventromedial prefrontal cortex (vmPFC) in response to the positive clips.

As Shammi and Stuss (1999) argued, the frontal lobe would appear to provide the “ideal substrate for integration at various levels of cognitive and affective functions.” The prefrontal cortex (PFC) seems intimately involved in the linguistic aspects of humor processing (Polimeni and Reiss, 2006). The subcortical dopaminergic reward system projects to the PFC (Schultz, 2000). In previous humor studies, women showed greater activation in the PFC for working memory and verbal functions than men (Azim et al., 2005), and men showed stronger activation in the dorsal prefrontal cortex (dPFC) (Kohn et al., 2011).

In our recent investigation, activation of the fronto-parietal lobe was associated with both EJs and AJs, suggesting that executive functions are involved in language and working memory processes (Chan and Lavallee, 2015). As noted earlier, EJs typically involve semantic extension, whereas AJs involve linguistic ambiguity. The PFC, particularly the frontopolar cortex (BA 10; also called the anterior prefrontal cortex or aPFC), is sensitive to tasks involved in monitoring information transfer and the processing of intentional states (Olsson and Ochsner, 2008). This region has also been implicated in cognitive flexibility and stability (Armbruster et al., 2012), monitoring action outcomes, selecting alternative tasks in response to a goal, and in deciding to switch tasks (Koechlin, 2011). Additionally, the aPFC, is also involved in processing novel, unpredictable events or multitasking event sequences (Koechlin et al., 1999, 2000), episodic and semantic retrieval, reasoning and problem solving (Christoff and Gabrieli, 2000).

The aPFC and dPFC are involved in the executive control of language, attention, and working memory functions (Doré et al., 2014) and play a role in more explicit cognitive processing (Forbes and Grafman, 2010). The aPFC (BA 10) is a key region for self-reflection in monitoring ongoing salient activation of working memory and shifting cognitive resources as needed; e.g., the flexibility of unexpected information processing in response to changing task demands (Koechlin et al., 1999, 2000). The dorsal PFC (dPFC, BA 9/8) can be separated into the dorsomedial PFC (dmPFC) and the dlPFC. The dmPFC is responsible for integrating and monitoring performance (e.g., decision making, selecting actions based on goals) and motivation (e.g., anticipating rewards), whereas the dlPFC is responsible for planning behaviors that reflect the complexity of control demands (Forbes and Grafman, 2010).

The feeling of amusement and humor appreciation has been associated with subcortical regions, including the amygdala (Mobbs et al., 2003; Watson et al., 2007; Chan et al., 2012; Chan and Lavallee, 2015), midbrain (Mobbs et al., 2003; Watson et al., 2007; Bekinschtein et al., 2011), insula (Moran et al., 2004), and PHG (Bartolo et al., 2006; Chan et al., 2012; Chan and Lavallee, 2015), as well as cortical regions, including OFC/vmPFC (Goel and Dolan, 2001; Chan et al., 2012; Chan and Lavallee, 2015). Women showed greater activation in the amygdala and insula than men in humor appreciation (Kohn et al., 2011). The amygdala is an integral component of the mesolimbic dopaminergic reward system in humor appreciation (Mobbs et al., 2003). The amygdala, which has reciprocal connections with the insula, plays a key role in emotional memory processes (Hamann, 2001). Reward-specific representations showed activation in the OFC (Sescousse et al., 2010). The interaction between emotion and working memory showed greater activation in the amygdala and OFC in women than in men (Koch et al., 2007). Social functions of the OFC integrate emotional processing and emotion regulation of self-monitoring, as well as evaluating rewards. In addition, the PHG may mediate information underlying positive emotion and is particularly active during successful encoding processes (Erk et al., 2003). fMRI studies of emotion and sex/gender differences in the PHG have yielded inconsistent results. Men relative to women showed stronger activation in the PHG during autobiographical memory retrieval of happy emotions (Piefke et al., 2005). Conversely, women showed greater density cluster activation in the PHG (Wager et al., 2003).

Jokes create associations through bridging-inference, exaggeration, and ambiguity techniques. The association may be incongruous and elicit the necessary surprise for a laughter response. The value of laughing is appreciated in every culture (Wild et al., 2006). Engagement of the left supplementary motor area (SMA) and pre-SMA are likely to reflect the motor aspects of the expressive laughter elicited by humor (Mobbs et al., 2003; Wild et al., 2006). The SMA may be expected to play a role in the differences in processing the laughter response between the sexes/genders.

### Research Purpose: The Neural Correlates of Interaction between Sex/Gender and Joke Type

This event-related fMRI study seeks to further advance our understanding of the sex/gender differences in humor comprehension, appreciation, and laughter by distinguishing the neural substrates of BJs, EJs, and AJs, and the corresponding non-joke baselines. The present study focused on eight regions of interest (ROIs) in the aPFC, dPFC, TPJ, OFC, amygdala, insula, PHG, and SMA. In addition, given the important role of the midbrain in modulation of affective amusement of humor, the study also included this region in the ROIs.

I hypothesized an interaction effect between sex/gender and joke types. I hypothesized that women would show greater activation in the TPJ than men in response to BJs, suggesting a response that is related to the ToM. I predicted that women would display increased activation in the anterior (frontopolar) prefrontal cortex (aPFC, BA 10) than men for the cognitive processing of humor in response to EJs and AJs, suggesting executive control in the language decoding and memory retrieval, whereas men would show stronger activation in the dPFC, related to executive control in the processes of goal-directed monitoring of performance, including cognitive reappraisal and cognitive self-control regulation. In the affective reward network, I expected that women would show greater activation in the OFC for BJs and in the amygdala and midbrain for EJs, whereas men would exhibit greater activation in the PHG for AJs. I also expected that women would exhibit increased activation in the SMA (BA 6) compared to men, suggesting the humor response in the explicit physical behavior of laughter.

## Materials and Methods

### Participants

Twenty-six healthy volunteers (13 women; mean age and *SD* = 24.27 ± 2.18; range, 22–29 years) participated in the study. All participants were native Mandarin speakers, right-handed, and had no history of neurological or psychiatric problems. Handedness was determined by the Edinburgh handedness inventory (Oldfield, 1971). Women had a mean age of 23.69 ± 1.70 and education averaged 16.38 ± 1.94 years. Men had a mean age of 24.46 ± 2.63 years and a mean education of 15.77 ± 1.30 years. The two groups differed neither in age, *t*(24) = 0.88, *p* = 0.095, nor in education, *t*(24) = 0.95, *p* = 0.073. The present study controlled for the effects of age and education between sexes/genders. Most participants in this study participated in the same experiment reported in Chan and Lavallee (2015). The study was carried out in accordance with the recommendations of the Research Ethics committee of National Taiwan University. All participants gave written informed consent in accordance with the Declaration of Helsinki.

### Stimuli

All jokes were written in Mandarin Chinese and designed to elicit humor-related cognitive and affective processing, followed by the laughter response. Each joke structure consisted of two components: the setup and the punch line. Verbal jokes were selected from the database of Chinese jokes (Cheng et al., 2013; Chan, 2014) or from websites. The 80 jokes in Mandarin Chinese included 30 BJs, 20 EJs, and 30 AJs. The corresponding baseline conditions were constructed by replacing the punch lines with neutral (unfunny) stories of matching length and punctuation, including 30 bridging-inference baseline stimuli (BS), 20 exaggeration baseline stimuli (ES), and 30 ambiguity baseline stimuli (AS). The criteria for selecting the stimuli were described in greater detail in Chan and Lavallee (2015). Few EJs were used because the current study did not include nonsense jokes and jokes related to psychiatric hospitals and patients.

The BJs were constructed using the inferring consequences logical mechanism. For example, in the funny condition, one joke reads:

Jack dreamed of being a writer since he was little. His dream comes true at the age of thirty when his book is finally published. One month later, Jack asks his friend, “Have you read my book yet?” his friend says: “Yes, and I bought one.” Jack happily responds: “Oh, that was you! Thanks!”

The unfunny condition (BS) reads “Jack happily responds: ‘Thanks for buying it.”

The EJs were constructed using the exaggeration logical mechanism. For example:

A restaurant was renowned for its stinginess. One day, a customer ordered a plate of soup. The waiter placed a plate on the table and kept the man waiting for a long while. The man signaled the manager to come and said “You have kept me waiting and you want me to have this wet plate?” The manager smiled and said: “Sir, this is your soup.”

In the unfunny condition (ES), the Boss smiled and said: “Sir, I will get a new one for you.”

Finally, the AJs were constructed using the juxtaposition logical mechanism. For example, one joke read:

In kindergarten, the kids were ready for a nap after going to the toilet. Jane suddenly rushed into the classroom and told the teacher: “Teacher, there are ants in the toilet.” The teacher realized that the kids had just recently learned the English word “ant,” so the teacher wanted to know how well Jane learned the word. The teacher asked: “So, how about the ant?” Jane: “The ants didn’t say anything.”

In the unfunny condition (AS), Jane says: “I have no idea.” (The ambiguity is more clear in the Chinese original: the Chinese phrase “mayi ruhe shuo” can mean both “how do you say ant?” and “what did the ants have to say?”)

To ensure that the jokes were valid stimuli, behavioral pilot studies were conducted prior to the fMRI experiment. The participants rated each trial on a 9-point scale. The mean and standard deviation for comprehensibility was 8.25 ± 0.83, indicating that all stimuli (joke and non-joke) were comprehensible to the participants. The mean funniness rating for all joke types was 6.06 ± 1.65. A one-way repeated-measures ANOVA performed on the participants’ funniness ratings was significant, *F*(5,235) = 226.67, *p* < 0.001, η_p_^2^ = 0.83, and Bonferroni *post hoc* tests revealed that the funny conditions were significantly funnier than the unfunny conditions.

### Experimental Paradigm

The stimuli were presented in an event-related fMRI paradigm. The experimental paradigm was presented using E-prime, and all stimuli were presented in black and white. The study examined the neural correlates of sex/gender differences across three joke types and the corresponding baseline stimuli (BJ-BS, EJ-ES, and AJ-AS). The participants were instructed to attentively watch all of the stimuli and were advised that some of the stimuli might be funny, whereas others might not be. In each trial, the participant was first shown the fixation for a jittered inter-stimulus interval (ISI), which was randomly varied among 2.1, 3.2, 5.6, and 7.9 s and counterbalanced across the stimulus types. Subsequently, the setup was shown once for 12 s, after which the punch line was delivered, lasting for 9 s. Finally, the participants provided a subjective funniness judgment by pressing one of four buttons on a keypad positioned under their right hand to indicate how funny the participant thought the stimuli was (1 = ‘not funny at all’ to 4 = ‘very funny’). The use of the hand for the button-press responses was counterbalanced in the scanner. A more detailed account of the design can be viewed in Chan and Lavallee (2015). There were five functional runs in total, and the first three TRs in each functional run were discarded to avoid T1 equilibrium effects. Each functional run lasted 8 min and 4 s, with a 2-min break between runs. The total duration of the experiment was approximately 48 min and 6 s per participant.

### Image Acquisition

The functional images were acquired on a 3-tesla MRI scanner (Megnetom, Skyra, Siemens) using a standard 32-channel head coil at National Chengchi University. The visual stimuli were presented to the participants on a projector. Every volume contained 32 transversal slices (4-mm-thick, no gap) in an interleaved order that were oriented parallel to the anterior and posterior commissures (AC-PC) and covered the whole brain, with a temporal resolution of 2 s using a T2*-weighted gradient echo spiral pulse sequence and the following acquisition parameters: echo time (TE) = 30 ms, repetition time (RT) = 2000 ms, and flip angle = 90°. The field of view (FOV) was 240 mm × 240 mm and the matrix size was 64 × 64, giving an in-plane spatial resolution of 3.75 mm. Each functional run to acquire 240 volumes took 8 min and 4 s. High-resolution T1-weighted structural images were also acquired using the 3D MPRAGE pulse sequence: TR = 1900 ms, TE = 3.30 ms, flip angle = 9°, 256 × 256 voxel matrix, FOV = 256 mm, 192 contiguous axial slices, thickness = 1.0 mm, and in-plane resolution: 1 mm × 1 mm × 1 mm.

### Image Analysis

All fMRI data were analyzed using Statistical Parametric Mapping software (SPM8; Wellcome Department of Cognitive Neurology, London, UK). Data from each participant were timing resliced, realigned, co-registered to the individual’s anatomical image, and normalized to the standard Montreal Neurological Institute (MNI, McGill University, Montreal, QC, Canada) T1 template. The statistical analyses were calculated on data that had been spatially smoothed using an 8-mm full-width-at-half-maximum (FWHM) Gaussian kernel with a high-pass filter (128-s cutoff period) to remove the low frequency artifacts. The functional images were corrected for differences in slice-acquisition time to the middle volume. The movement was no more than 3 mm in any plane.

After preprocessing, each participant was analyzed for his or her responses to the jokes compared to the non-joke baseline stimuli for each condition using a general linear model (GLM). For the event-related analysis, the functions corresponding to the onset of different event types were constructed and convolved with a canonical hemodynamic response function (HRF) and its temporal derivative. In a first level analysis (single subject analyses), the different event types (jokes and non-jokes) were defined, and the parameter estimates for each regressor were calculated for each voxel. The stimuli were treated as individual events for analysis and modeled for the punch line using a canonical HRF. To increase the statistical sensitivity and to remove the motion-related artifacts, the present study also included six motion parameters as regressors of no interest in the first level GLM.

It is common for neuroimaging studies to compare groups based on an *a priori* hypothesis of previous research (Kaiser et al., 2009; Rippon et al., 2014). A region of interest (ROI) statistical analysis was performed for a specific *a priori* hypothesis (Poldrack et al., 2008; Kaiser et al., 2009; Bluhm, 2013a,b; Fine, 2013; Rippon et al., 2014). Anatomical ROI maps were generated using WFU PickAtlas Tool software that generates ROI masks (Maldjian et al., 2003). The resulting mask of humor processing was associated with brain regions in the predefined ROI, specifically the analyses focused on 8 ROIs in the aPFC (BA 10), dPFC (BA 9/8), TPJ (BA 39), amygdala, PHG, insula (BA 13), OFC (BA 11/47), and SMA (BA 6).

The parameter estimates from the first-level analysis were entered into a second-level (random effects) analysis using the flexible factorial design to test inferences during between group analyses. The between group analysis was then conducted using a two-way mixed analysis of variance (ANOVA) design, which allowed us to parse the main effect of joke type (BJ-BS versus EJ-ES versus AJ-AS), main effect of sex/gender (women versus men, and men versus woman), and interactions between the joke type and sex/gender. In addition, given the important role of the midbrain in modulation of affective amusement of humor, the study also included a region in the ROIs for each simple main effect. The threshold of activation of the predefined ROIs were set at a voxel-wise *p* < 0.05 FWE (family-wise error rate) for multiple comparisons with five contiguous voxels using a small volume correction (SVC) and using a 10-mm sphere on the coordinates of interest.

In addition, to confirm any between group differences, a time-series analysis was conducted to determine between group differences in the magnitude of percent changes in the blood-oxygenation level dependent response (BOLD) signal (effect size) (Surguladze et al., 2005). Previous studies demonstrated to report effect size using percent signal change (PSC), parameter estimates (beta or con) and Cohen’s *d* (Pernet, 2014). The present study used the PSC to describe sex/gender effect magnitude in the time-series analysis and mean beta values in between group analyses. The event-related responses to a given event were plotted in peri-stimuli time bins. The plot in terms of the fitted response and peri-stimulus time histograms (PSTH) was the average response to an event with a mean signal ± SE for each peri-stimulus time bin. The time course of the hemodynamic responses shows far more variability in timing and shape between sexes/genders and enabled us to visualize significant signal changes for brain regions by extracting the peak voxels of the regions from the beta values. The present study extracted the average time courses for the different types of jokes between the sexes/genders.

Finally, one previous meta-analysis has investigated whether brain regions differ in activation using both between group analysis and within group variances (Joel et al., 2015). Therefore, the present study also performed an analysis of within group variances for women and men. In addition, further analyses were performed on randomly assigned participants in two random groups in order to provide an additional check on whether the sex/gender differences that were found could be due to chance (Frost et al., 1999).

## Results

### Behavioral Results

The participants were requested to rate the funniness of a joke on a 4-point scale (1 = not funny at all, 2 = not funny, 3 = funny, 4 = very funny) during the scanning procedure. The mean funniness rating for all joke types was 3.01 ± 0.44 and for unfunny jokes was 1.80 ± 0.41. A one-way repeated-measures ANOVA of the participants’ funniness ratings was significant, *F*(5,125) = 184.25, *p* < 0.001, η_p_^2^ = 0.88, and Bonferroni *post hoc* tests revealed that three funny conditions were significantly funnier than the three unfunny conditions. A main effect of sex/gender, *F*(1,24) = 0.298, *p* > 0.05, was not significant. The interaction among joke types (BJ-BS, EJ-ES, and AJ-AS) and sex/gender analyses, *F*(2,48) = 0.087, *p* > 0.05, was not significant.

### Sex/Gender Differences in Brain Activation: Between Group Analyses

For the between group analyses, an interaction between joke type and sex/gender was revealed in the left medial frontal gyrus, left insula, right PHG, left middle frontal gyrus, left SMA, and right amygdala (**Table 1**). A joke type main effect was observed in the left insula, right middle frontal gyrus, right TPJ, and right SMA. The sex/gender main effect revealed that women showed greater activation in the right aPFC, left PHG, right cingulate gyrus, and left insula than men, whereas men showed greater activation in the left dPFC than women. A *post hoc* test showed significant simple main effects for each of the different types of jokes between the sexes/genders (**Table 2**).

**Table 1:** Between group comparisons of brain regions associated with main effects and interactions among joke types and sex/gender.

Region	BA	Voxels	MNI coordinates			*Z*-score
			*x*	*y*	*z*	
**Main effect of joke type**						
Insula	13	106	–45	–4	4	4.23
Middle frontal gyrus	47/10	65	51	41	–2	3.78
Temporoparietal junction (TPJ/IPL)	39	16	51	–67	40	3.77
Supplementary motor area (SMA)	6	57	3	11	67	3.69
**Main effect of sex/gender (women versus man)**						
Anterior prefrontal cortex (aPFC)	10	58	33	59	–2	5.89
Parahippocampal gyrus	19	91	–30	–52	–8	4.33
Cingulate gyrus	24	84	9	2	40	4.03
Insula	13	37	–45	–13	–8	3.56
**Main effect of sex/gender (man versus women)**						
Dorsal prefrontal cortex (dPFC)	8/9	56	–9	47	49	5.09
**Interaction effect (joke type × sex/gender)**						
Medial frontal gyrus	6	143	0	–10	67	4.09
Insula	13	105	–42	–28	16	4.02
Parahippocampal gyrus	34	41	24	2	–17	4.00
Middle frontal gyrus (dPFC)	9/8	56	–57	17	28	3.93
Supplementary motor area (SMA)	6	112	–6	–10	73	3.86
Amygdala	–	39	18	–4	–11	3.70

*The activation threshold of main effects and interaction was set at p < 0.05 FWE (family-wise error rate) corrected for multiple comparisons.*

**Table 2:** Between group comparisons in brain regions differentially activated for the simple main effects and within group variances in frequency and percentage.

Priori region		BA	Voxels	Side	MNI coordinates			*Z-*score	W	M	W%	M%
					*x*	*y*	*z*					
**Bridging-inference jokes (BJ)**											
Women versus men	aPFC	10	26	R	27	59	1	3.50	10	6	76.92%	46.15%
		10	36	L	–18	56	–2	3.41	11	5	84.62%	38.46%
	TPJ	39	34	R	45	–76	13	3.36	7	4	53.85%	30.77%
	PHG	30	112	R	21	–49	1	3.85	7	4	53.85%	30.77%
		19	129	L	–24	–55	–8	3.85	6	3	46.15%	23.08%
	Insula	13	118	L	–39	–28	16	4.04	7	2	53.85%	15.38%
	OFC	11	37	L	–30	38	–11	3.88	10	9	76.92%	69.23%
	SMA	6	63	L	–6	–10	70	3.92	7	4	53.85%	30.77%
		6	95	R	12	–10	67	3.67	6	4	46.15%	30.77%
Men versus women	dPFC	8	15	L	–9	47	49	3.71	8	12	61.54%	92.31%
**Exaggeration jokes (EJ)**											
Women versus men	aPFC	10	54	R	33	59	–2	5.30	6	4	46.15%	30.77%
		10	38	L	–27	53	–5	4.15	9	4	69.23%	30.77%
	Amygdala	–	5	R	21	–10	–11	3.54	10	5	76.92%	38.46%
	*Midbrain*	–	23	R	9	–13	–8	3.35	6	3	46.15%	23.08%
	PHG	19	32	L	–36	–49	–8	3.32	7	6	53.85%	46.15%
	Insula	13	61	L	–45	–13	–8	4.07	10	5	76.92%	38.46%
Men versus women	dPFC	8/9	7	L	–9	41	52	3.02	4	7	30.77%	53.85%
**Ambiguity jokes (AJ)**											
Women versus men	aPFC	10	29	R	27	59	1	3.65	7	5	53.85%	38.46%
Men versus women	dPFC	8	72	R	51	11	46	4.81	2	7	15.38%	53.85%
		9	58	L	0	56	34	4.11	7	10	53.85%	76.92%
	PHG	34	26	R	24	2	–17	3.66	5	10	38.46%	76.92%
**Women**											
BJ versus EJ versus AJ	dPFC	9	84	L	–57	17	28	5.39	8		61.54%	
	TPJ/IPL	39	18	R	54	–70	16	3.67	11		84.62%	
		40	73	R	57	–46	49	4.41	9		69.23%	
	Amygdala	–	73	R	24	–4	–17	5.28	10		76.92%	
	PHG	34	68	R	21	2	–14	5.29	9		69.23%	
	Insula	13	131	R	45	2	–2	5.13	9		69.23%	
	SMA	6	125	L	0	11	67	5.04	12		92.31%	
**Men**												
BJ versus EJ versus AJ	dPFC	6	82	L	–3	–13	64	4.10		8		61.54%
	TPJ/IPL	39	18	R	51	–67	40	3.89		11		84.62%
	Insula	13	33	R	39	–13	19	3.28		6		46.15%

*The activation threshold for simple main effects was set at p < 0.05 FWE (family-wise error rate) for multiple comparisons within five contiguous voxels using in eight predefined ROIs for each type of jokes. The other ROI of midbrain regions are indicated by italics. aPFC, anterior (frontopolar) prefrontal cortex; dPFC, dorsal prefrontal cortex; TPJ, temporoparietal junction; PHG, parahippocampal gyrus; OFC, orbitofrontal cortex; SMA, supplementary motor area; W, frequency activation in women; M, frequency activation in men; W%, percentage activation in women; M%, percentage activation in men.*

#### Sex/Gender Differences for Bridging-Inference Jokes

**FIGURE 2:**
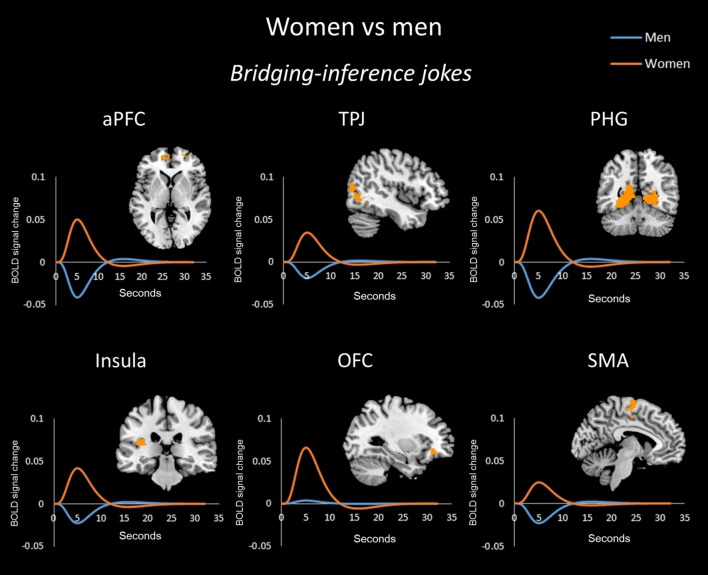
**Bridging-inference jokes (BJs) between the sexes/genders.** Blood-oxygenation level dependent response (BOLD) signal activation for BJs versus baselines and time-series analysis in women versus men. Women showed greater activation than men in the anterior (frontopolar) prefrontal cortex (aPFC), temporoparietal junction (TPJ), parahippocampal gyrus (PHG), insula, orbitofrontal cortex (OFC), and supplementary motor area (SMA).

In the bridging-inference jokes (BJ-BS) condition, women showed greater activation than men in the bilateral aPFC, right TPJ, bilateral PHG, left insula, left OFC, and bilateral SMA (**Figure 2**), whereas men showed greater activation than women in the left dPFC (**Figure 4**).

#### Sex/Gender Differences for Exaggeration Jokes

**FIGURE 3:**
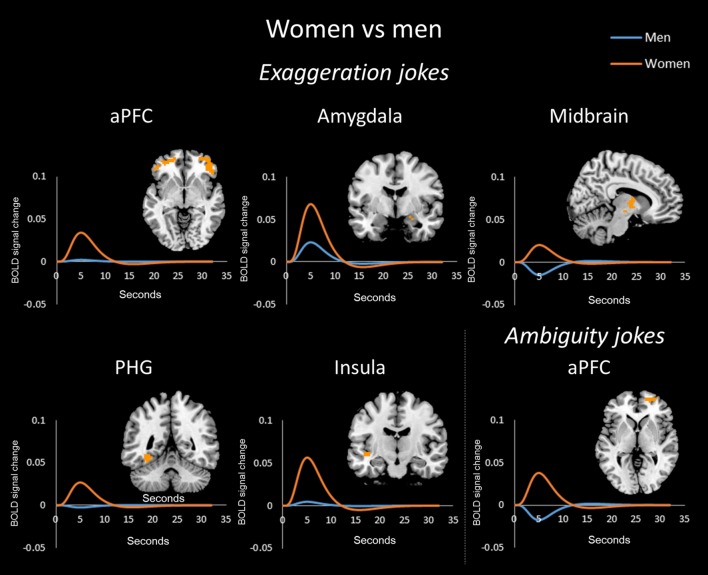
**Exaggeration jokes (EJs) and ambiguity jokes (AJs) between the sexes/genders.** BOLD signal activation for the EJs versus baselines and time-series analysis in women versus men. Women showed greater activation than men in the anterior (frontopolar) prefrontal cortex (aPFC), amygdala, midbrain, parahippocampal gyrus (PHG), and insula for EJs. In addition, BOLD signal activation for AJs versus baseline and time-series analysis in women versus men. Women showed greater activation than men in the aPFC for AJs.

**FIGURE 4:**
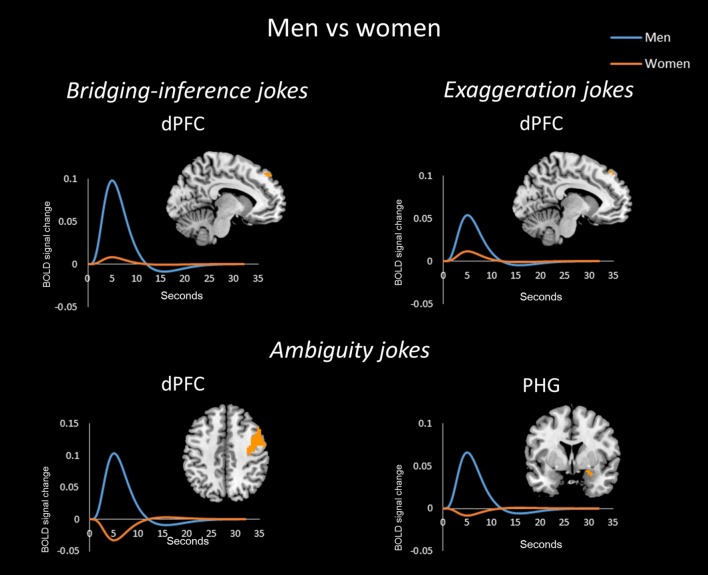
**Activation in men for all three joke types.** For all joke types, men displayed more activation than women in the dorsal prefrontal cortex (dPFC) according to the mean time courses of the hemodynamic response for the activated voxels. Additionally, men showed greater activation in the parahippocampal gyrus (PHG) for AJs.

For the exaggeration jokes (EJ-ES) condition, women showed greater activation than men in the bilateral aPFC, right amygdala, left PHG, and left insula. Women also showed greater activation than men in the right midbrain (**Figure 3**). Men showed greater activation than women in the left dPFC (**Figure 4**).

#### Sex/Gender Differences for Ambiguity Jokes

In the ambiguity jokes (AJ-AS) condition, women showed greater activation than men in the right aPFC (**Figure 3**), whereas men showed greater activation than women in the bilateral dPFC and right PHG (**Figure 4**).

#### Joke Type Differences for Women

For women, differences in the processing of the three joke types were found in the left dPFC, right TPJ/IPL, right amygdala, right PHG, right insula, and left SMA.

#### Joke Type Differences for Men

For men, differences in the processing of the three joke types were found in the left dPFC, right TPJ/IPL, and right insula.

### Between Group Differences in BOLD Signal Changes: Time-Series Analysis

To further analyze the sex/gender differences in the magnitude of changes in BOLD signals (i.e., effect size) associated with each type of joke, a time-series analysis was performed to determine the response to the different contrast types in each region, which was then averaged across participants.

The hemodynamic response peaked in amplitude at approximately 6 s after the stimulus onset (i.e., at the estimated time point of peak BOLD response), followed by the slow BOLD response to the stimulus (modeled neuronal activation). The average BOLD response returned to the baseline level at approximately 12 s and was followed by a longer shallow undershoot.

The time-series analysis identified BOLD signal increases in a given region for each joke type, which was compared to negligible BOLD signal decreases for jokes that were not funny (Azim et al., 2005). The orange line in each graph shows the averaged response to each stimulus for all women and the blue line shows the average response for men. Women exhibited greater activation than men in the aPFC, TPJ, PHG, insula, OFC, and SMA in response to BJs (**Figure 2**). Women exhibited activation in the TPJ with BJs, whereas men demonstrated decreased activation. Additionally, women showed greater activation in the OFC with BJs, whereas men showed little activation. In terms of EJs and AJs, women showed greater activation than men in the aPFC, amygdala, midbrain, PHG, and insula for EJs, as well as in the aPFC for AJs (**Figure 3**). Conversely, men showed greater activation than women in the dPFC for all three type jokes and greater activation in the PHG for AJs (**Figure 4**).

### Within Group Variance Analysis

The present study examined the coordinates of a given ROI in the first-level analysis in the results of each participants using a SVC and using a 10-mm sphere on the ROI. Results for all participants that met this ROI activation criterion were summed by group, and the frequencies and percentage of ROI activation for women and men are listed in Table 2.

**FIGURE 5:**
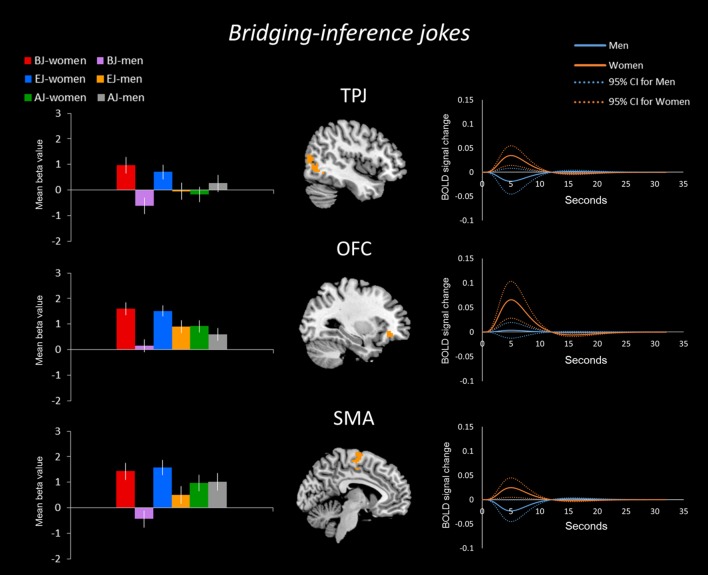
**BOLD signal activation for BJs by women and men – comparison. (Left)** The sex/gender differences in the BOLD signal activation for the three types of jokes. Regarding the sex/gender differences for the BJ condition, women showed stronger mean differences in betas than men in the TPJ, OFC, and SMA. **(Right)** The time-series analysis (solid line) for the BJ condition indicated that the BOLD signal change was bigger in these regions for women than men; the curves of the 95% confidence interval (dotted line) reveal the within group variance for women and men.

**FIGURE 6:**
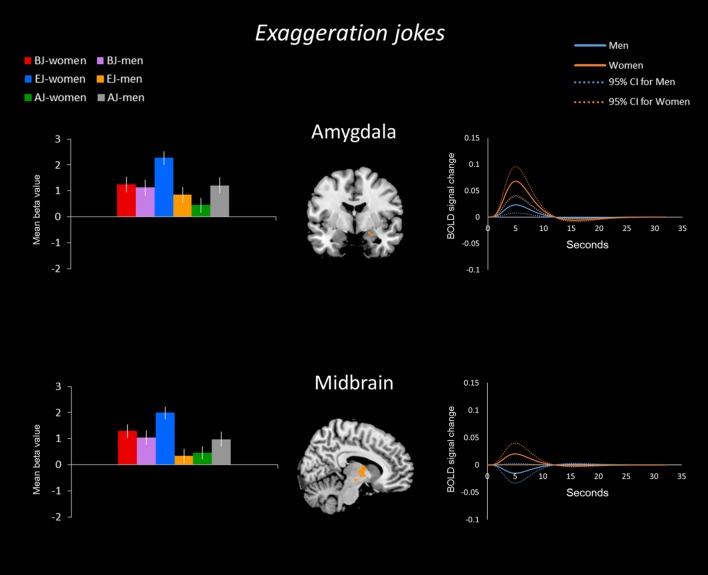
**BOLD signal activation for EJs by women and men – comparison. (Left)** The sex/gender differences in the BOLD signal activation for the three types of jokes. Regarding the sex/gender differences for the EJ condition, women showed stronger mean differences in betas than men in the amygdala and midbrain. **(Right)** The time-series analysis (solid line) for the EJ condition indicated that the BOLD signal change was bigger in these regions for women than men; the curves of the 95% confidence interval (dotted line) reveal the within group variance for women and men.

**FIGURE 7:**
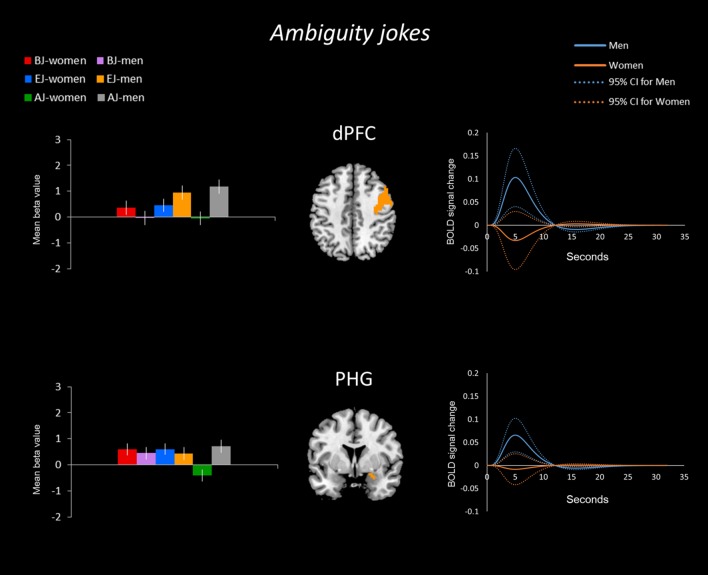
**BOLD signal activation for AJs by men and women – comparison. (Left)** The sex/gender differences in the BOLD signal activation for the three types of jokes. Regarding the sex/gender differences for the AJ condition, men showed stronger mean differences in betas than women in the dPFC and PHG. **(Right)** The time-series analysis (solid line) for the AJ condition indicated that the BOLD signal change was bigger in these regions for men than women; the curves of the 95% confidence interval (dotted line) reveal the within group variance for men and women.

To explore the variance within each of the two groups, the present study carried out an analysis of the 95% confidence interval through the PSTH of time-series analysis using the signal changes for brain regions by extracting the peak voxels of the regions from the beta values. The curves of within group variance results are shown in **Figures 5–7.** When the area of the confidence interval for the PSTH is larger, it indicates greater within group variance.

### Random Group Analysis

A random-grouping analysis was employed to provide an additional check on whether the sex/gender differences that were found could be due to chance. Participants were initially grouped into male and female groups of 13 participants each. Pseudorandomly assigned groups were then constructed by systematically altering the ratio of men and women in each group. To construct groups at each ratio, participants were randomly selected and exchanged between the two groups. For example, in the first round, one participant from each group was randomly chosen and the two were exchanged so that the formerly ‘male’ group now included one woman and 12 men and the formerly ‘female’ group included one man and 12 women (Mode A). A mixed ANOVA of groups and joke types based on the aforementioned data was then performed. This procedure was repeated three times. Participants were then returned to the original all-male and all-female groups. Next, the process was repeated but with two women randomly selected and moved to the ‘male’ group and two men randomly selected and moved to the ‘female’ group, creating a second ‘mode’ of the sex/gender ratio (Mode B). Once again, the same statistical analysis was performed three times. Then, a third mode was created by exchanging three participants from each group, and so on, until six participants from each group had been exchanged (almost 50% of the members each group). This resulted in a total of 18 mixed ANOVA analyses (see **Table 3**).

**Table 3:** Random group analysis by pseudorandom exchange modes.

Mode	Number of randomly selected participants exchanged	Percentage of participants exchanged	Number of analyses	Number of significant interactions
A	1	7.69%	3	0
B	2	15.38%	3	0
C	3	23.08%	3	1
D	4	30.77%	3	0
E	5	38.46%	3	0
F	6	46.15%	3	0

Total			18	1

Only one of the results, occurring when three participants (23.08%) were exchanged, was significantly different. In this analysis, an interaction of joke types and random-grouping was found in the brain region of the right IFG (BA 46/47, *Z* = 4.36, 20 voxels) with a threshold at a voxel-wise *p* < 0.05 FWE using a SVC and using a 10-mm sphere on the ROI.

## Discussion

The present study used event-related fMRI to identify the neural substrates of sex/gender differences associated with humor comprehension (incongruity and resolution) and the perception of humor response, including humor appreciation (feeling of amusement), and humor expression (laughter) for three types of verbal jokes by comparing BJs, EJs, and AJs, as well as their corresponding non-joke baselines. Previous fMRI studies investigating humor showed that women and men display differences in both cognitive and affective processing (Azim et al., 2005; Kohn et al., 2011). Starting with the findings for the cognitive processing of humor, according to Azim et al. (2005), women show PFC activation (e.g., dlPFC and IFG) more than men when viewing funny cartoons versus unfunny baselines, whereas men did not activate any region more than women. According to Kohn et al. (2011), both women (e.g., vlPFC, IFG) and men (e.g., dlPFC, IFG) showed stronger activation in the PFC. These two studies appear inconsistent in the patterns of PFC activation between sexes/genders. The present study found sex/gender differences in the aPFC in women and the dPFC in men for all three types of semantic verbal jokes. These two prefrontal areas (the aPFC and dPFC) mediate the cognitive operations required to comprehend particular joke types.

The present study more precisely identified sex/gender differences in the aPFC, particularly in the frontopolar cortex (BA 10). In all types of jokes, women exhibited greater activation in the aPFC than men. The aPFC has been implicated in cognitive flexibility and stability (Armbruster et al., 2012), executive functions (Koechlin et al., 2000), and integrating the outcomes of two or more separate cognitive operations (Ramnani and Owen, 2004). The findings of the present study suggest that women may recruit and activate more verbal functions and deploy greater episodic memory retrieval for humor integration than men. Additionally, the present findings in women may suggest that the functions of the lateral frontopolar cortex (MFG and SFG; lFPC; BA 10) include assisting with the specific cognitive processes required to resolve incongruities related to semantic ambiguity, exaggerative distortion, and semantic gaps, all tasks that require working memory operations.

The present study also found that men showed greater activation than women in the prefrontal modulatory regions (dmPFC and dlPFC) for all three types of jokes. The dPFC is involved in integrating and monitoring self-regulation, motivation (dmPFC), cognitive reappraisal and action selection (dlPFC) (Forbes and Grafman, 2010), and in facilitating the encoding of information through the use of context (Maher et al., 1995). Upon detecting incongruities, men appeared to respond with greater activation of cognitive control processes in the dPFC, goal-directed and effortful cognitive processing (e.g., schema shifting), regulation of disambiguation, meaning extension, and backward-inference processing in order to resolve the incongruities.

Previous studies related to emotions have indicated that women show a heightened experience of emotions, particularly those that are negative (Whittle et al., 2011). The present findings relate to the positive emotion of mirth or amusement and may offer insight into the mechanisms underlying sex/gender differences in the experience of amusement. The present study found that women exhibited greater activation than men in a temporoparietal-mesocortical-motor network comprising the right TPJ (BA 39), left OFC (BA 11), and bilateral SMA (BA 6) for jokes requiring bridging-inferences (BJs). Women also showed greater activation than men in the frontal-mesolimbic network comprising the bilateral aPFC (BA 10), right amygdala and midbrain for exaggerative jokes (EJs), whereas men showed greater activation than women in the frontal-paralimbic network comprising the bilateral dPFC (BA 9/8) and right PHG in response to AJs.

One important distinguishing feature of the BJs, in comparison with EJs and AJs, is that the joke endings were unexpected and the participants were required to fill in the unstated implications, which requires the reader to ‘get the joke’ by contextual-bridging and making inferences. As these inferences involved attributing intentions and beliefs to others, the comprehension of BJs thus requires ‘ToM’ processing (Chan and Lavallee, 2015). In the present study, women responded to BJs, with greater TPJ (BA 39) activation than men, suggesting that women made more inferences about others’ attributing intentions to resolve the incongruities. Once the unexpected incongruity was resolved, activation in the OFC occurred, presumably related to the evaluation and regulation of rewards. The OFC has been implicated in social-affective amusement and we surmise that the resolution of the joke triggered a sense of superiority, which in turn elicited the response of laughter in the SMA. Previous studies have suggested that SMA activity likely reflects the motor aspects of the expressive laughter elicited by humor (Mobbs et al., 2003; Wild et al., 2006). Additionally, Fried et al. (1998) found that a patient laughed when the SMA was stimulated. We note, however, that Amir et al. (2013) found SMA activation for both humorous and non-humorous conditions, suggesting the alternative possibility that the SMA might function in cognitive processing (e.g., monitoring conflict) rather than in motor functioning. Sex/gender differences in SMA activation related to laughter or associated with cognitive processing should be investigated further.

An important contribution of this study relates to sex/gender differences in the neural correlates of affective response to particular verbal jokes. Previous humor fMRI studies have suggested that women show greater neurobiological correlates to humor appreciation than men (Azim et al., 2005; Kohn et al., 2011; Vrticka et al., 2013). However, the present study identified differences between men and women in affective amusement that were specific to particular types of joke. Women exhibited greater neural correlates of social affective amusement in the cortex (OFC) for BJs and in the limbic system (amygdala, midbrain and insula) for EJs. Conversely, men showed greater activation than women in the paralimbic system (PHG) for AJs. We discuss each in turn.

Activation of the OFC may be related to the processing of reward coding and motivational functions (Schultz et al., 2000). The OFC is one of key structures in the cortical-basal ganglia reward circuit and plays a central role in evaluating the value, magnitude, and probability of rewards (Haber and Knutson, 2010). Moreover, social positive amusement stimuli (e.g., comedy) have been associated with greater OFC activation (Britton et al., 2006; Chan and Lavallee, 2015). The OFC is important for social emotional regulation and evaluating rewards in humor processing and plays a key role in modulating limbic reactivation (Saddoris et al., 2005). The present study shows greater activation of this region for women responding to BJs.

This study also found that women showed greater activation than men in the amygdala and midbrain in response to EJs, implying a possibly greater reward salience. The amygdala is a key component in regulating the reward circuit (Haber and Knutson, 2010). Animal studies have suggested that the function of mesolimbic dopamine, which is implicated in mesolimbic dopaminergic input from the ventral tegmental area (VTA) in the midbrain for the reward pathway, represents attention to novel, salient or rewarding events that require an effortful response (Schultz et al., 1993; Berridge and Robinson, 1998). EJs create a distorting incongruity which results in humor. Women may enjoy a greater sense of superiority based on the implied depreciation of a target in the humor episode when the exaggeration incongruity is resolved.

Whereas men show less activation than women in the OFC, amygdala, and midbrain in response to BJs and EJs, men demonstrated *greater* activation than women in the PHG in response to AJs. The PHG is important for recognition and reward predictions (Chan et al., 2012; Chan, 2015; Chan and Lavallee, 2015).

The present study performed a between group analysis by ROIs in humor processing. The present study also performed a random grouping analysis with the two participant groups (men and women). The procedure showed empirically the number of non-significant findings and the non-expected amount of sex/gender variability appearing by chance. The results of this random grouping analysis indicate significant sex/gender differences in responses to different types of jokes. Finally, the present study performed a within group variance analysis. This analysis found differences in within group variance in response to different types of jokes for women and men.

In summary, the present study built on the tri-component theory of humor (Chan, 2015) to provide an important advance in our understanding of the differences in neural mechanisms used by men and women in processing different types of jokes. For all joke types, women showed greater activation in the anterior (frontopolar) prefrontal gyrus (aPFC, BA 10) than men, whereas men exhibited greater activation than women in the dPFC (BA 9/8). Women showed greater activation than men in the *temporoparietal–mesocortical-motor* network associated with BJs, whereas women exhibited more activation than men in the *frontal-mesolimbic* network associated with EJs. Conversely, men showed greater activation than women in the *frontal-paralimbic* network associated with AJs. The findings of the present study suggest that there are sex/gender differences in the neural mechanisms supporting cognitive, affective, and laughter processing for different types of verbal jokes. Future studies could examine the functional effective connectivity between these regions in each of the sexes/genders in response to each of the three types of jokes.

These findings may contribute to the development of a broader theory of the humor-related differences between women and men. As noted above, attempts have been made to explain such differences in terms of both biological and social–cultural factors. It is our hope that the present findings of specific sex/gender-based differences in responses at the neural level will prove useful to future discussions of exactly *how* and *why* women and men differ when it comes to humor.

## Author Contributions

The author designed and conducted the experiment, analyzed the data, provided the findings, and wrote the paper.

## Conflict of Interest Statement

The author declares that the research was conducted in the absence of any commercial or financial relationships that could be construed as a potential conflict of interest.
